# Supplementary material for: XLF acts as a flexible connector during non-homologous end joining
Source: eLife. 2020 Dec 8;9:e61920. doi: 10.7554/eLife.61920 (PMC7744095; doi:10.7554/eLife.61920)
Supplement: Supplementary file 1. — Table containing replicate number, number of molecules tracked, number of SR complex formation events detected, average SR complex formation rate, and the standard error of the mean for the rate for each experimental condition. [file elife-61920-supp1.docx]

**Supplementary File 1**

| **Protein Construct** | **# Replicates** | **# Molecules** | **Event #** | **Ave Formation Rate (s^-1^)** | **SEM (s^-1^)** |
| --- | --- | --- | --- | --- | --- |
| Buffer | 3 replicates | 778 | 15 | 6.4x10^-5^ | 1.0x10^-5^ |
| wt XLF | 3 replicates | 691 | 267 | 1.7x10^-3^ | 3.4x10^-4^ |
| XLF^1-245+KBM^ | 4 replicates | 1076 | 13 | 4.6x10^-5^ | 1.3x10^-5^ |
| H10-XLF  Flag-Avi XLF | 2 replicates | 306 | 132 | 3.1x10^-3^ | 1.9x10^-4^ |
| H10-XLF^ΔTailΔKBM^  Flag-Avi-XLF | 5 replicates | 1391 | 222 | 6.2x10^-4^ | 1.2x10^-4^ |
| H10-XLF^ΔTailΔKBM^ | 5 replicates | 998 | 16 | 8.6x10^-5^ | 3.9x10^-5^ |
