# Supplementary material for: XLF acts as a flexible connector during non-homologous end joining
Source: eLife. 2020 Dec 8;9:e61920. doi: 10.7554/eLife.61920 (PMC7744095; doi:10.7554/eLife.61920)
Supplement: Supplementary file 2. — Table containing figure panel reference, type of data, replicate number, data shown, and reference to details of any statistical analysis for each data-based figure panel. [file elife-61920-supp2.docx]

**Supplementary File 2**

| **Figure** | **Type** | **Replicate #** | **Shown** | **Statistics** |
| --- | --- | --- | --- | --- |
| 1C | Time course end joining assay | 3 | Representative gel image | N/A |
| 1D | End point end joining titration assay | 2 | Representative gel image | N/A |
| 2B | Time course end joining assay | 3 | Representative gel image | N/A |
| 3B | smFRET trajectory | 691 | Representative trajectory | N/A |
| 3C | smFRET trajectory | 691 | Representative trajectory | N/A |
| 3D | FRET efficiency histogram | 3 | Histogram curves (include all replicates) | N/A |
| 3E | FRET efficiency histogram | 3 | Histogram curves (include all replicates) | N/A |
| 3F | FRET efficiency histogram | 4 | Histogram curves (include all replicates) | N/A |
| 3G | Plot of SR complex formation rates | 3, 3, 4* | Individual replicate rates, mean rate, and 95% CI | N/A |
| 4A | Time course end joining assay | 3 | Representative gel image | N/A |
| 4C | Time course end joining assay | 4 | Representative gel image | N/A |
| 4E | Time course end joining assay | 3 | Representative gel image | N/A |
| 5B | DNA pulldown immunoblots | 3 | Representative immunoblots | N/A |
| 6B | Cellular NHEJ GFP-reporter assay | 6 | Mean GFP+ frequencies with Std Dev error | Details in Fig 6 caption |
| Fig 1 fig supp 1B | XLF immunoblot | 4 | Representative anti-XLF immunoblot | N/A |
| Fig 2 fig supp 1A | Time course end joining assay | 3 | Representative gel image | N/A |
| Fig 2 fig supp 1B | Bar graph of XLF T_m_ values | 2 | Mean T_m_ with min and max error bars | Details in Figure 2-figure supplement 1B caption |
| Fig 3 fig supp 1A | smFRET trajectory | 778 | Representative trajectories | N/A |
| Fig 3 fig supp 1B | smFRET trajectory | 1076 | Representative trajectories | N/A |
| Fig 4 fig supp 2A | Time course end joining assay | 4 | Representative gel image | N/A |
| Fig 4 fig supp 2B | XLF heterodimer exchange assay | 3 | Representative anti-XLF and anti-His immunoblots | N/A |
| Fig 4 fig supp 2D | SEC-MALS data plot | 1*** | Rayleigh ratio peaks and corresponding molecular masses | N/A |
| Fig 4 fig supp 3A | Plot of SR complex formation rates | 2, 5, 5** | Individual replicate rates, mean rate, and 95% CI | Details in Figure 4-figure supplement 3A caption |
| Fig 4 fig supp 3B | smFRET trajectory | 998 | Representative trajectory | N/A |
| Fig 4 fig supp 3C | smFRET trajectory | 1391 | Representative trajectory | N/A |
| Fig 4 fig supp 3D | smFRET trajectory | 306 | Representative trajectory | N/A |
| Fig 6 fig supp 1A | Expression of Flag-XLF constructs in mESCs | 1 | anti-Flag and anti-Actin immunoblots | N/A |
| Fig 6 fig supp 1B | Recombinant XLF and XLF heterodimers | 1 | SDS-PAGE gel image | N/A |
| Fig 6 fig supp 1C | Recombinant XLF and tandem dimer XLF | 1 | SDS-PAGE gel image | N/A |
|  |  |  |  |  |
| * 3 replicates each for the ΔXLF + buffer and ΔXLF + wt XLF. 4 replicates for the ΔXLF + XLF^1-245+KBM^ condition | | | |  |
| **2 replicates for the ΔXLF + Flag-Avi-XLF/H10-XLF. 5 replicates each for the ΔXLF + Flag-Avi-XLF/H10-XLF^ΔTailΔKBM^ and ΔXLF + H10- XLF^ΔTailΔKBM^ conditions | | | |  |
| ***1 replicate for each sample shown on shared plot | | | |  |
